# Hepatitis C Virus Core Protein Abrogates the DDX3 Function That Enhances IPS-1-Mediated IFN–Beta Induction

**DOI:** 10.1371/journal.pone.0014258

**Published:** 2010-12-08

**Authors:** Hiroyuki Oshiumi, Masanori Ikeda, Misako Matsumoto, Ayako Watanabe, Osamu Takeuchi, Shizuo Akira, Nobuyuki Kato, Kunitada Shimotohno, Tsukasa Seya

**Affiliations:** 1 Department of Microbiology and Immunology, Hokkaido University Graduate School of Medicine, Sapporo, Japan; 2 Department of Tumor Virology, Okayama University Graduate School of Medicine, Dentistry, and Pharmaceutical Sciences, Okayama, Japan; 3 Laboratory of Host Defense, WPI Immunology Frontier Research Center, Research Institute for Microbial Diseases, Osaka University, Suita, Japan; 4 Research Institute, Chiba Institute of Technology, Narashino, Japan; Duke University Medical Center, United States of America

## Abstract

The DEAD box helicase DDX3 assembles IPS-1 (also called Cardif, MAVS, or VISA) in non-infected human cells where minimal amounts of the RIG-I-like receptor (RLR) protein are expressed. DDX3 C-terminal regions directly bind the IPS-1 CARD-like domain as well as the N-terminal hepatitis C virus (HCV) core protein. DDX3 physically binds viral RNA to form IPS-1-containing spots, that are visible by confocal microscopy. HCV polyU/UC induced IPS-1-mediated interferon (IFN)-beta promoter activation, which was augmented by co-transfected DDX3. DDX3 spots localized near the lipid droplets (LDs) where HCV particles were generated. Here, we report that HCV core protein interferes with DDX3-enhanced IPS-1 signaling in HEK293 cells and in hepatocyte Oc cells. Unlike the DEAD box helicases RIG-I and MDA5, DDX3 was constitutively expressed and colocalized with IPS-1 around mitochondria. In hepatocytes (O cells) with the HCV replicon, however, DDX3/IPS-1-enhanced IFN-beta-induction was largely abrogated even when DDX3 was co-expressed. DDX3 spots barely merged with IPS-1, and partly assembled in the HCV core protein located near the LD in O cells, though in some O cells IPS-1 was diminished or disseminated apart from mitochondria. Expression of DDX3 in replicon-negative or core-less replicon-positive cells failed to cause complex formation or LD association. HCV core protein and DDX3 partially colocalized only in replicon-expressing cells. Since the HCV core protein has been reported to promote HCV replication through binding to DDX3, the core protein appears to switch DDX3 from an IFN-inducing mode to an HCV-replication mode. The results enable us to conclude that HCV infection is promoted by modulating the dual function of DDX3.

## Introduction

The retinoic acid inducible gene-I (RIG-I) and the melanoma differentiation-associated gene 5 (MDA5) encode cytoplasmic RNA helicases [1]–[3] that signal the presence of viral RNA through the adaptor, IPS-1/Mitochondrial antiviral signaling protein (MAVS)/Caspase recruitment domain (CARD) adaptor inducing interferon (IFN)-beta (Cardif)/Virus-induced signaling adaptor (VISA) to produce IFN-beta [4]–[7]. IPS-1 is localized to the mitochondrial outer membrane through its C-terminus [6]. Increasing evidence suggests that the DEAD-box RNA helicase DDX3, which is on the X chromosome, participates in the regulation of type I IFN induction by the RIG-I pathway.

**Figure 1:**
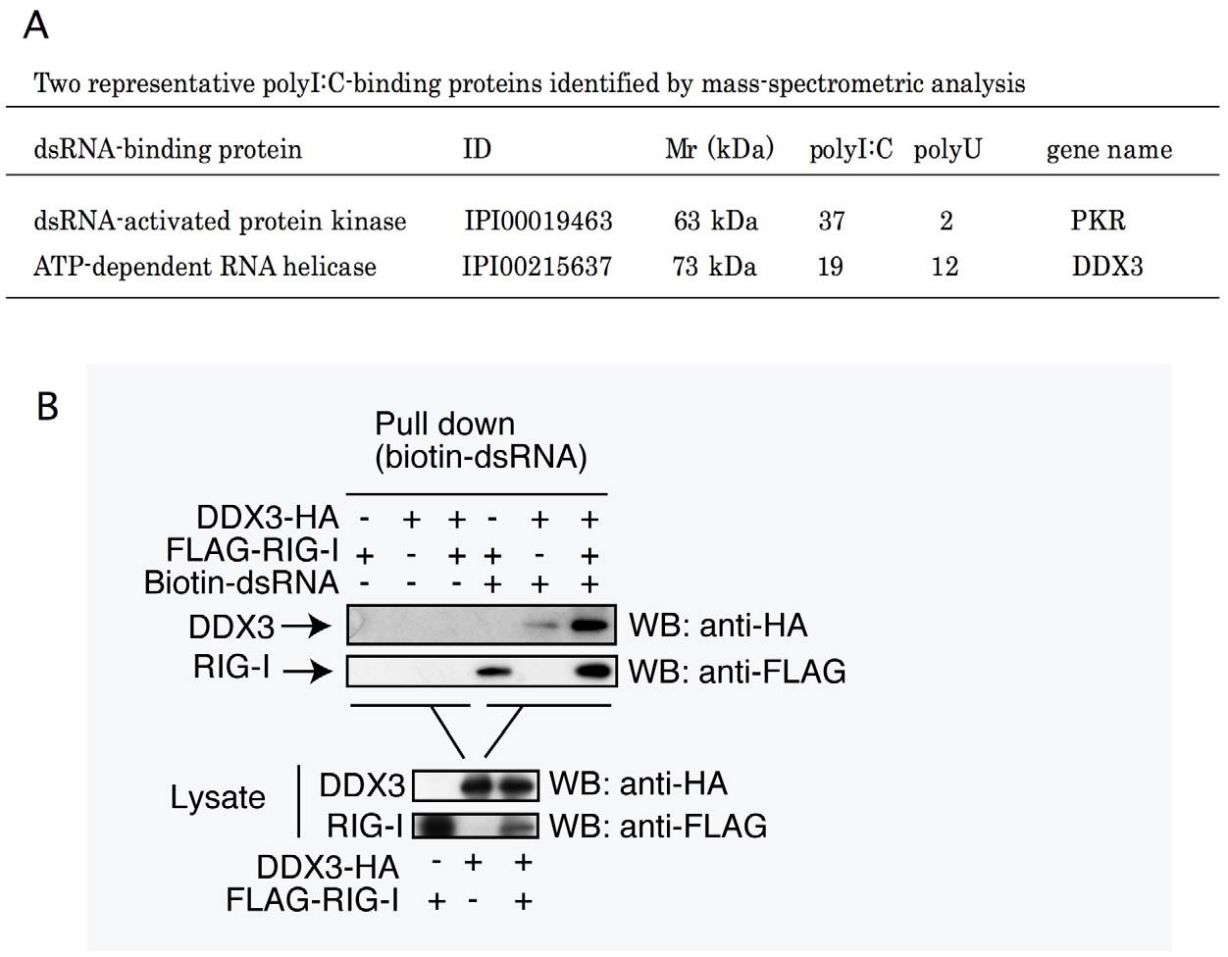
DDX3 is a RNA-binding protein. (A) DDX3 is a polyU- and polyI:C-binding protein. Mass spectrometry analyses indicated that DDX3 binds polyI:C- and polyU-Sepharose, although PKR binds polyI:C but not polyU. The rough data from MASCOT and one representative of six trials are shown. (B) DDX3 binds dsRNA, RIG-I and HCV core protein. Expression vectors for Flag-tagged RIG-I and HA-tagged DDX3 were transfected into HEK293 cells using lipofectamine 2000. Twenty-four hours after the transfection, extract from transfected cells were mixed with biotin-conjugated dsRNA. RNA-protein complex were recovered by pull-down assay using streptavidin-Sepharose. The protein within the pull-down fraction was analyzed by western blotting. The results are representative of two independent experiments.

DDX3 acts on the IFN-inducing pathway by a complex mechanism. Early studies reported that DDX3 up-regulates IFN-beta induction by interacting with IKKepsilon [8] or TBK1 [9] in a kinase complex. Both TBK1 and IKKepsilon are IRF-3-activating kinases with NF-kappaB- and IFN-inducible properties. DDX3 has been proposed to bind IKKepsilon, and IKKepsilon is generated after NF-kappaB activation [10]. Yeast two-hybrid studies demonstrated that DDX3 binds IPS-1, and both are constitutively present prior to infection (Fig. 1). Ultimately, DDX3 forms a complex with the DEAD-box RNA helicases RIG-I and MDA5 [11], which are present at only low amounts in resting cells, and are up-regulated during virus infection. Previously we used gene silencing and disruption, to show that the main function of DDX3 is to interact with viral RNA and enhance RIG-I signaling upstream of NAP1/TBK1/IKKepsilon [11]. Hence, DDX3 is involved in multiple pathways of RNA sensing and signaling during viral infection.

DDX3 resides in both the nucleus and the cytoplasm [12], and has been implicated in a variety of processes in gene expression regulation, including transcription, splicing, mRNA export, and translation [13]. A recent report suggested that the N-terminus of hepatitis C virus (HCV) core protein binds the C-terminus of DDX3 (Fig. S1) [14], , and this interaction is required for HCV replication [16]. Although DDX3 promotes efficient HCV infection by accelerating HCV RNA replication, the processes appear independent of its interaction with the viral core protein [15]. HCV seems to co-opt DDX3, and require DDX3 for replication. In addition, the association between DDX3 and core protein implicates DDX3 in HCV-related hepatocellular carcinoma progression [17]. Therefore, DDX3 could be a novel target for the development of drugs against HCV [18].

A number of reports have demonstrated the formation of the DDX3-core protein complex in the cytoplasm, but the functional relevance of DDX3-core protein interaction is not known. In this report, we show evidence that the HCV core protein participates in suppression of DDX3-augmented IPS-1 signaling for IFN-β induction. Several possible functions of DDX3 are discussed, focusing on its core protein association and IPS-1-regulatory properties.

## Materials and Methods

### Cell culture and reagents

HEK293 cells and HEK293FT cells were maintained in Dulbecco's Modified Eagle's low or high glucose medium (Invitrogen, Carlsbad, CA) supplemented with 10% heat-inactivated FCS (Invitrogen) and antibiotics. Huh7.5 cells were maintained in MEM (Nissui, Tokyo, Japan) supplemented with 10% heat-inactivated FCS. Hepatocyte sublines with HCV replicon (O cells) and without replicon (Oc cells) were established as described previously [19]. O cells with core-less subgenomic replicon (sO cells) were also generated in Dr. Kato's laboratory [16], [19]. RIG-I −/− mouse embryonic fibroblasts (MEF) were gifts from Drs. Takeuchi and Akira [1]. Anti-FLAG M2 monoclonal Ab and anti-HA polyclonal Ab were purchased from Sigma. A mitochondria marker (Mitotracker) and Alexa Fluor®-conjugated secondary antibodies were purchased from Molecular probe. Anti-HCV core mAb (C7-50) [20] and anti-human DDX3 pAb were from Affinity BioReagents, Inc and Abcam, Cambridge MA, respectively.

### Plasmids

DDX3 cDNA encoding the entire ORF was cloned into pCR-blunt vector using primers, DDX3N F-Xh (CTC GAG CCA CCA TGA GTC ATG TGG CAG TGG AA) and DDX3C R-Ba (GGA TCC GTT ACC CCA CCA GTC AAC CCC) from human lung cDNA library. To make an expression plasmid, HA tag was fused at the C-terminal end of the full length DDX3 (pEF-BOS DDX3-HA). pEF-BOS DDX3 (1-224aa) vector was made by using primers, DDX3 N-F-Xh and DDX3D1 (GGA TCC GGC ACA AGC CAT CAA GTC TCT TTT C). pEF-BOS DDX3-HA (225-662) was made by using primers, DDX3D2-3 (CTC GAG CCA CCA TGC AAA CAG GGT CTG GAA AAA C) and DDX3C R-Ba. To make pEF-BOS DDX3-HA (225-484) and pEF-BOS DDX3-HA (485-663), the primers, DDX3D2 R-Ba (GGA TCC AAG GGC CTC TTC TCT ATC CCT C) and DDX3D3 F-Xh (CTC GAG CCA CCA TGC ACC AGT TCC GCT CAG GAA AAA G) were used, respectively. HCV core expressing plasmids, pcDNA3.1 HCVO core or JFH1 core, were previously reported by N. Kato (Okayama University Japan) [16]. Another 1b genotype of the core was cloned from a HCV patient in Osaka Medical Center (Osaka) according to the recommendation of the Ethical Committee in Osaka. We obtained written informed consent from each patient for research use of their samples. Reporter and internal control plasmids for reporter gene assay are previously described [21], [22].

### Preparation of HCV polyU/UC RNA

The HCV genotype 1b polyU/UC RNA (from 9421 to 9480, Accession number: EU867431) [23] was synthesized by T7 RNA polymerase *in vitro*. The template dsDNA sequences were; Forward: TAA TAC GAC TCA CTA TAG GGT TCC CTT TTT TTT TTT CTT TTT TTT TTT TTT TTT TTT TTT TTT TTT CTC CTT TTT TTT TC, Reverse: GAA AAA AAA AGG AGA AAA AAA AAA AAA AAA AAA AAA AAA AAA AGA AAA AAA AAA AGG GAA CCC TAT AGT GAG TCG TAT TA. The synthesized RNA was purified by TRIZOL reagent (Invitrogen). cDNA of HCV 3′ UTR region was amplified from total RNA of O cells using primers HCV-F1 and HCV-R1, and then cloned into pGEM-T easy vector. The primer set sequences were HCV-F1: CTC CAG GTG AGA TCA ATA GG and HCV-R1: CGT GAC TAG GGC TAA GAT GG. RNA was synthesized using T7 and SP6 RNA polymerases. Template DNA was digested by DNase I, and RNA was purified using TRIZOL (Invitrogen) according to manufacturer's instructions.

### RNAi

Knockdown of DDX3 was carried out using siRNA, DDX3 siRNA-1: 5′-GAU UCG UAG AAU AGU CGA ACA-3′, siRNA-2: 5′-GGA GUG AUU ACG AUG GCA UUG-3′, siRNA-3: 5′-GCC UCA GAU UCG UAG AAU AGU-3′ and control siRNA: 5′-GGG AAG AUC GGG UUA GAC UUC-3′. 20 pmol of each siRNA was transfected into HEK293 cells in 24-well plate with Lipofectamin 2000 according to manufacture's protocol. Knockdown of DDX3 was confirmed 48 hrs after siRNA transfection. Experiments were repeated twice for confirmation of the results.

### Reporter assay

HEK293 cells (4×10^4^ cells/well) cultured in 24-well plates were transfected with the expression vectors for IPS-1, DDX3 or empty vector together with the reporter plasmid (100 ng/well) and an internal control vector, phRL-TK (Promega) (2.5 ng/well) using FuGENE (Roche) as described previously [23]. The p-125 luc reporter containing the human IFN-beta promoter region (−125 to +19) was provided by Dr. T. Taniguchi (University of Tokyo, Tokyo, Japan). The total amount of DNA (500 ng/well) was kept constant by adding empty vector. After 24 hrs, cells were lysed in lysis buffer (Promega), and the *Firefly* and *Renella* luciferase activities were determined using a dual-luciferase reporter assay kit (Promega). The *Firefly* luciferase activity was normalized by *Renella* luciferase activity and is expressed as the fold stimulation relative to the activity in vector-transfected cells. Experiments were performed three times in duplicate (otherwise indicated in the legends).

### PolyI:C or polyU/UC stimulation

PolyI:C was purchased from GE Healthcare company, and solved in milliQ water. For polyI:C treatment, polyI:C was mixed with DEAE-dextran (0.5 mg/ml) (Sigma) in the culture medium, and the cell culture supernatant was replaced with the medium containing polyI:C and DEAE-dextran. Using DEAE-dextran, polyI:C is incorporated into the cytoplasm to activate RIG-I/MDA5.

HCV 3′ UTR poly U/UC region (PU/UC) RNA (0∼50 ng/well), which is synthesized *in vitro* by T7 RNA polymerase, transfected into HEK293 cells in 24-well plate by lipofectamin 2000 (Invitrogen) with other plasmids. Cells were allowed to stand for 24∼48 hrs and HCV RNA-enhancing activation of IFN-beta promoter was assessed by reporter assay.

### Immunoprecipitation (i.p.)

HEK293FT cells were transfected in a 6-well plate with plasmids encoding DDX3, IPS-1, RIG-I or MDA5 as indicated in the figures. 24 hrs after tranfection, the total cell lysate was prepared by lysis buffer (20 mM Tris-HCl [pH 7.5] containing 125 mM NaCl, 1 mM EDTA, 10% Glycerol, 1% NP-40, 30 mM NaF, 5 mM Na_3_Vo_4_, 20 mM IAA and 2 mM PMSF), and the protein was immunoprecipitated with anti-HA polyclonal (SIGMA) or anti-FLAG M2 monoclonal Ab (SIGMA). The precipitated samples were resolved on SDS-PAGE, blotted onto a nitrocellulose sheet and stained with anti-HA (HA1.1) monoclonal (SIGMA), anti-HA polyclonal or anti-FLAG M2 monoclonal Ab.

### Pull-down assay

The pull-down assay was performed according to the method described in Saito T et al. [24]. Briefly, the RNA used for the assay was purchased from JBioS, Co. Ltd (Saitama, Japan). The RNA sequences are (sense strand) AAA CUG AAA GGG AGA AGU GAA AGU G, (antisense strand)CAC UUU CAC UUC UCC CUU UCA GUU U. The biotin is conjugated at U residue at the 3′ end of antisence strand (underlined). Biotinylated double-stranded (ds)RNA were incubated for 1 hr at 25°C with 10 µg of protein from the cytoplasmic fraction of cells that were transfected with Flag-tagged RIG-I and HA-tagged DDX3 expressing vectors. The mixture was transferred into 400 µl of lysis buffer containing 25 µl of streptavidine Sepharose beads, rocked at 4°C for 2 h, collected by centrifugation, washed three times, resuspended in SDS sample buffer.

### Proteome analysis of RNA-binding proteins

RNA-binding proteins were identified by affinity chromatography and Mass spectrometry. Briefly, cell lysate was prepared from human HEK293 or Raji cells as will be described elsewhere (Watanabe and Matsumoto, manuscript submitted for publication). The lysate was first applied to polyU-Sepharose and then the pass-through fraction was applied to PolyI:C-Sepharose. The eluted proteins were analyzed on Mass spectrometry using the MASCOT software.

### Confocal analysis

HCV replicon-positive (O) or –negative (Oc) cells were plated onto cover glass in a 24-well plate. In the following day, cells were transfected with indicated plasmids using Fugene HD (Roch). The amount of DNA was kept constant by adding empty vector. After 24 hrs, cells were fixed with 3% of paraformaldehyde in PBS for 30 minutes, and then permeabilized with PBS containing 0.2% of Triton X-100 for 15 min. Permeabilized cells were blocked with PBS containing 1% BSA, and were labeled with anti-Flag M2 mAb (Sigma) or anti-HA pAb (Sigma) in 1% BSA/PBS for 1 hr at room temperature [25]. In some cases, endogenous proteins were directly stained with anti-core (C7-50) mAb (Affinity BioReagents, Inc) or anti-DDX3 pAbs (Abcam, Cambridge MA). The cells were then washed with 1% BSA/PBS and treated for 30 min at room temperature with Alexa-conjugated antibodies (Molecular Probes). Thereafter, micro-cover glass was mounted onto slide glass using PBS containing 2.3% DABCO and 50% of glycerol. The stained cells were visualized at ×60 magnification under a FLUOVIEW (Olympus, Tokyo, Japan).

## Results

### DDX3 binds RNA species

We have performed proteome analyses of RNA-binding fractions in human dendritic cell lysate eluted from polyU and polyI:C Sepharose. 127 cytoplasmic proteins were reproducibly identified as polyI:C-binding proteins (Watanabe and Matsumoto, unpublished data). Four of them are DEAD/H box helicases. In this setting, we found DDX3 is a RNA-binding protein (Fig. 1A). DDX3 in cell lysate bound both polyU and polyI:C, while the control PKR bound only to polyI:C.

Using biotinylayed dsRNA, RNA-binding properties of DDX3 and RIG-I were tested by pull-down assay. DDX3 or RIG-I protein was co-precipitated with dsRNA in HEK293 cells expressing either alone of DDX3 or RIG-I (Fig. 1B). Strikingly, higher amounts of DDX3 and RIG-I were precipitated with dsRNA in cells expressing both proteins (Fig. 1B). This, taken together with previous results [11], [14], [16], indicates that DDX3 assembles in some RNA, RIG-I, IPS-1 and HCV core protein in its C-terminal domain (Fig. S1).

### PolyU/UC but not replicon enhances IFN-β induction via IPS-1/DDX3

**Figure 2:**
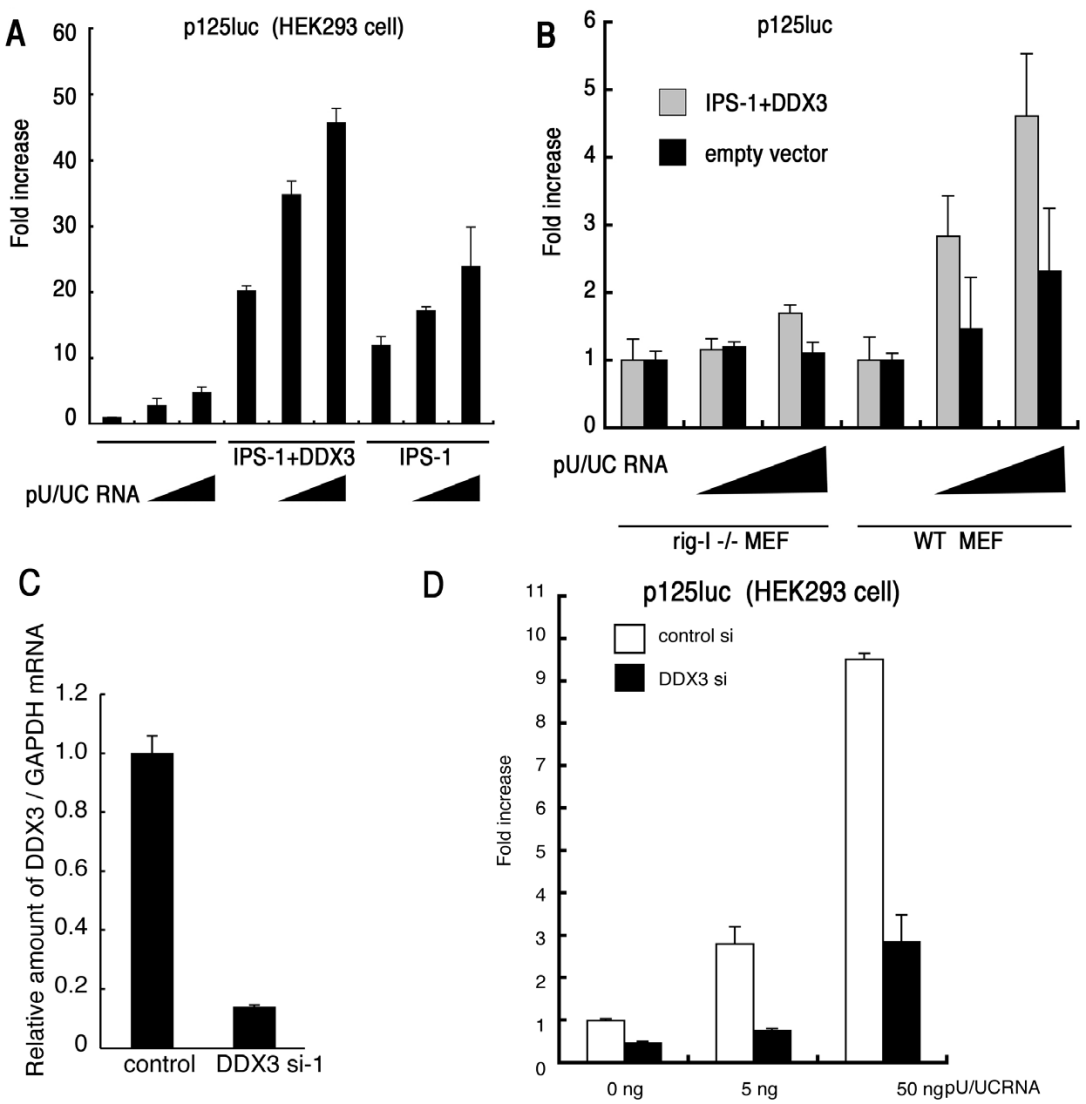
DDX3 is a positive regulator of IPS-1-mediated IFN promoter activation. (A) IFN-β induction by polyU/UC is augmented by DDX3. IPS-1 (100 ng), DDX3 (100 ng) and p125luc reporter (100 ng) plasmids were transfected into HEK293 cells in 24-well plates with or without the HCV 3′ UTR poly U/UC region (PU/UC) RNA (0, 25 or 50 ng/well), synthesized *in vitro* by T7 RNA polymerase. HCV RNA-enhancing activation of IFN-beta promoter was assessed by reporter assay in the presence or absence of the DDX3-IPS-1 complex. (B) RIG-I is essential for the DDX3/IPS-1-mediated IFN-promoter activation. MEF from wild-type and RIG-I −/− mice were transfected with plasmids of IPS-1, DDX3 and p125luc as in panel A, and stimulated with polyU/UC (0, 25 or 50 ng/well). Reporter activity was determined as in panel A. (C) Knockdown of DDX3. Negative control or DDX3 targeting siRNA (20 pmol), DDX3 si-1, was transfected into HEK293 cells, and after 48 hrs, expression of endogenous DDX3 mRNA was examined by real-time RT-PCR. DDX3 si-1-mediated down-regulation of the DDX3 protein was also confirmed by Western blotting (data not shown). (D) DDX3 enhances RIG-I-mediated IFN-beta promoter activation induced by polyU/UC. DDX3 si-1 or control siRNA was transfected into HEK293 cells with reporter plasmids (100 ng). After 48 hrs, cells were stimulated with polyU/UC (5∼50 ng/ml) with lipofectamin 2000 reagent for 6 hrs, and activation of the reporter p125luc was measured. The results are representative of at least two independent experiments, each performed in triplicate.

A polyU/UC sequence is present in the 3′-region of the HCV genome, and serves as a ligand for RIG-I in IPS-1 pathway activation [23]. We produced the polyU/UC RNA and tested its IFN-beta-inducing activity in the presence or absence of DDX3 and IPS-1 (Fig. 2A). HCV polyU/UC promoted IPS-1-mediated IFN-beta induction, and this was further enhanced by forced expression of DDX3/IPS-1 (Fig. 2A). Similar results were obtained with wild-type mouse embryonic fibroblasts (MEF) (Fig. 2B). We also investigated whether DDX3 enhanced IPS-1-mediated IFN-β promoter activation in a RIG-I −/− MEF background (Fig. 2B). In IPS-1/DDX3-expressing MEF cells, polyU/UC IFN-induction was almost totally abrogated by the lack of RIG-I, suggesting that the trace RIG-I protein in the IPS-1 complex is required for DDX3 enhancement of the polyU/UC-mediated IFN response.

DDX3 mRNA (Fig. 2C) and protein [11] were depleted in HEK293 cells by gene silencing with si-1 siRNA, so this was used for DDX3 loss-of-function analysis. Control or DDX3-silinced cells were transfected with increasing amounts of polyU/UC and IFN-beta promoter activation was determined by luciferase assay. DDX3 loss-of-function resulted in a decrease of promoter activation by intrinsic polyU/UC (Fig. 2D). The result was confirmed with cells over-expressing RIG-I and exogenous polyI:C stimulation. HEK293 cells were transfected with a plasmid for the expression of RIG-I and stimulated with polyI:C, an activator of the IPS-1 pathway (Fig. S2A). IFN-beta reporter activation was suppressed in si-1-treated cells that expressed RIG-I, since polyI:C lots often contain short size duplexes that can activate RIG-I [26]. In addition, DDX3 augmented the IFN-beta response in cells expressing MDA5/IPS-1 (Fig. S2B). Thus, DDX3 was also crucial for IPS-1-mediated IFN-beta promoter activation.

**Figure 3:**
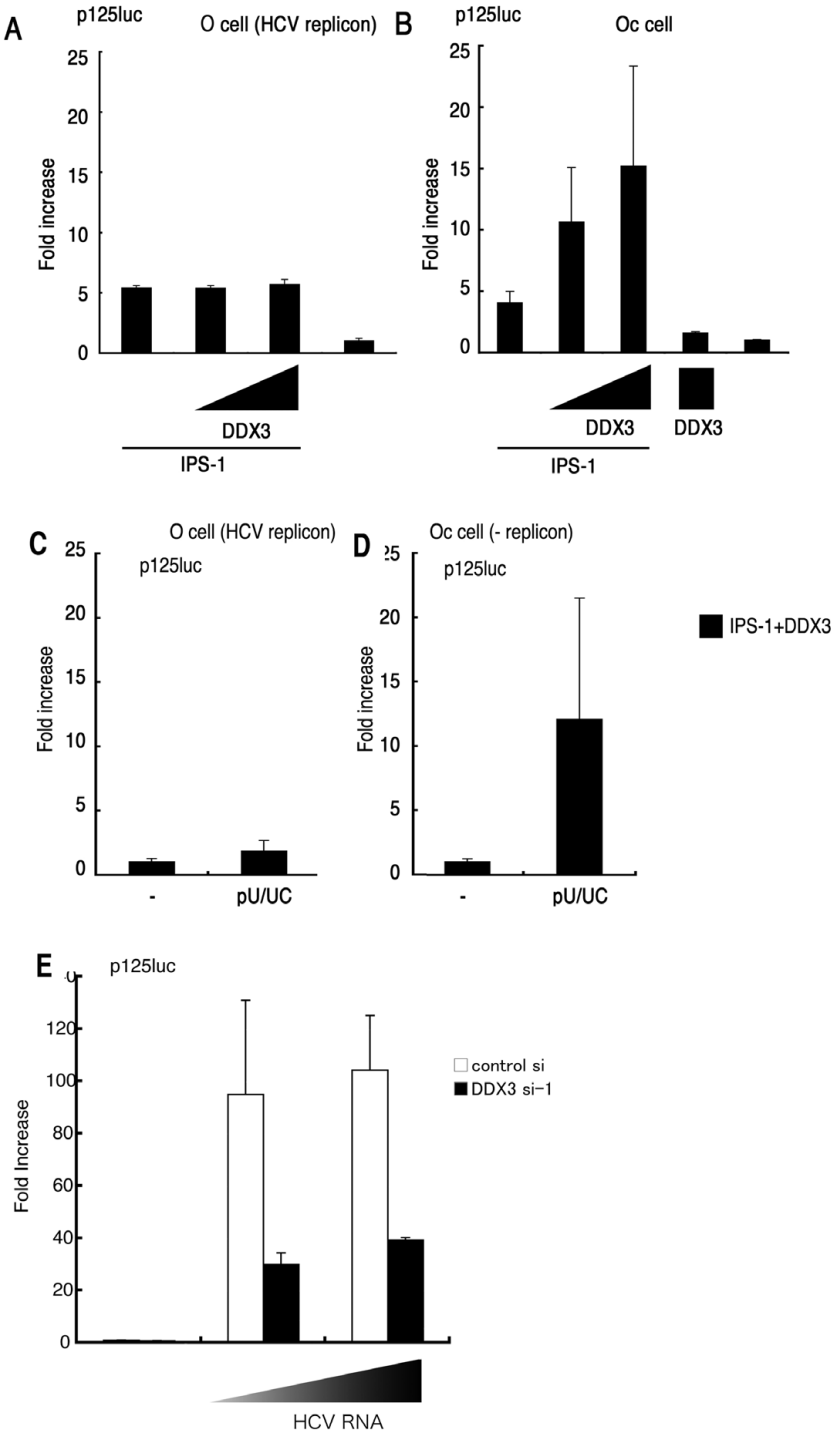
The HCV replicon suppresses IPS-1/DDX3-mediated augmentation of IFN promoter activation. (A,B) O cells with the HCV replicon fail to activate an IFN-β reporter in response to IPS-1/DDX3. O cells contain the full-length HCV replicon, and Oc cells do not [16]. O cells (A) or Oc cells (B) were transfected with IPS-1, DDX3 or p125luc reporter plasmids. At timed intervals (24 hrs), reporter activity was determined as in Fig. 2. (C,D) The HCV replicon suppresses IFN-promoter activation by polyU/UC. O cells and Oc cells expressing IPS-1 and DDX3 were stimulated with polyU/UC. At 48 hrs, reporter activity was determined as in panel A. (E) DDX3 is required for enhanced activation of IFN-beta promoter by O cell HCV 3′UTR. HCV 3′ UTR cDNA was amplified by RT-PCR from RNA extracted from O cells containing full-length HCV replicon. The HCV 3′ UTR RNA was synthesized *in vitro* using T7 RNA polymerase. DDX3 siRNA or control siRNA was transfected into HEK293 cells with the p125luc reporter. After 24 hrs, cells were transfected with HCV RNA, and incubated for 24 hrs. The IFN-beta promoter activation was assessed by luciferase reporter assay. One representative of at least three independent experiments, each performed in triplicate, is shown.

We next determined whether the HCV replicon triggers IPS-1/DDX3 IFN promoter activation, using human hepatocyte lines with the HCV replicon (O cells) or without it (Oc cells). In O cells with the HCV replicon, IPS-1/DDX3 expression showed minimal enhancement of IFN-beta promoter activation (Fig. 3A), while in control Oc cells with no replicon, DDX3 facilitated IFN-beta promoter activation (Fig. 3B). Similarily, an augmented IFN promoter response to polyU/UC was observed in control Oc cells, but not in O cells (Figs. 3C and 3D). HCV RNA was prepared from O cells, and its ability to activate the IFN-beta reporter was tested in HEK293 cells (Fig. 3E). The HCV RNA of O cells had a high potency to induce reporter activation, and this activity was largely abrogated by si-1 siRNA treatment. Therefore, DDX3 augments IPS-1-mediated IFN-beta promoter activation in hepatocyte O cells, and HCV RNA, presumably the 3′UTR, participates in this induction. However, no IFN-beta reporter activation was detected in O cells which harbor HCV replicon. Therefore, an unidentified viral factor appeared to participate in suppressing virus RNA-mediated IFN-beta induction, which occurred in O cells overexpressing DDX3/IPS-1.

### HCV core protein inhibits IPS-1 signaling through DDX3

**Figure 4:**
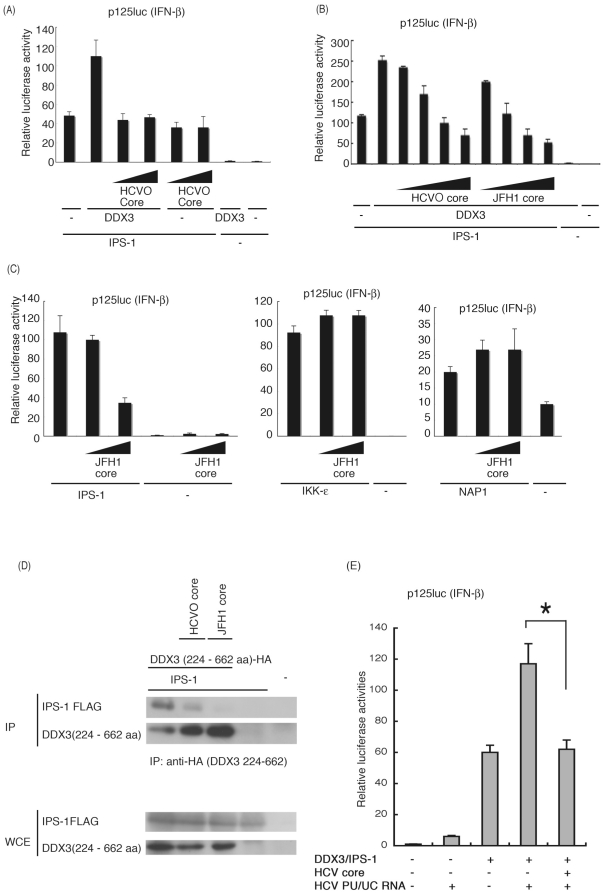
HCV core protein inhibits DDX3 promotion of IPS-1-mediated IFN-beta induction. (A) Expression plasmids for IPS-1 (100 ng), DDX3 (200 ng) and/or HCVO core (50 or 100 ng) were transfected into HEK293 cells in 24-well plates with reporter plasmids, and reporter activity was examined. (B) Expression plasmids for IPS-1 (100 ng), DDX3 (100 ng), and/or HCVO or JFH1 core (10, 25, 50 or 100 ng) were transfected into HEK293 cells, and reporter gene expression was analyzed. (C) IPS-1-, IKKepsilon- or NAP1-expressing plasmids were transfected into HEK293 cells with HCV JFH1 core-expressing plasmids (25 or 100 ng), for reporter gene analysis. (D) Plasmids for expression of FLAG-tagged IPS-1 (400 ng), HA-tagged DDX3 partial fragment (400 ng) and HCVO or JFH1 core (400 ng) were transfected into HEK293FT cells. 24 hrs later cells were lyzed and the lysate was incubated with anti-HA Ab for immunoprecipitation. The DDX3 (224-662)-bound IPS-1 was blotted onto a sheet and probed with anti-Flag Ab. Whole cell lysate was also stained with anti-tag Abs. (E) IPS-1 (100 ng), DDX3 (100 ng), JFH1 core (50 ng) and/or p125 luc reporter (100 ng) plasmids were transfected with HEK293 cells, with HCV 3′UTR poly-U/UC (PU/UC) RNA (25 ng), synthesized *in vitro*. Cell lysates were prepared after 24 hrs, and luciferase activities measured. One representative of at least three independent experiments is shown except for panel D, which is a representative of two sets of the experiments.

What HCV proteins participate in IFN-beta induction was tested in a pilot study using protein expression analysis. We found that expression of HCV core protein as well as NS3/4A led to suppression of IFN-beta reporter activity in Oc cells (data not shown). The HCV core protein physically binds DDX3 [14], [16], and co-localizes with DDX3 in the cytoplasm of HeLa cells transfected with HCV core protein [14]. Furthermore, we showed that DDX3 binds IPS-1, which resides on the mitochondrial outer membrane, and assembles into RNA-sensing receptors. Since some populations of the HCV core protein localize on the mitochondrial outer membrane [27], we tested if HCV core protein affects IPS-1 signaling by binding to DDX3. The cDNAs for HCV core proteins, genotype 1b (HCVO) and 2a (JFH1) [16], were co-transfected into HEK293 together with IPS-1, DDX3, and reporter plasmids, and core protein interference with IPS-1/DDX3-mediated IFN-beta promoter activation was examined. We found that the core proteins of HCVO and JFH1 suppressed IPS-1/DDX3-augmented IFN-beta-induction in a dose-dependent manner (Fig. 4A and 4B). Without DDX3 transfection, core protein had no effect on IPS-1-mediated IFN-beta promoter activation (Fig. 4A). JFH1 core slightly more efficiently inhibited IPS-1/DDX3-augmented IFN-beta-induction than HCVO core (Fig. 4B).

Although some endogenous DDX3 was present in the cytoplasm without DDX3 transfection, only IPS-1 transfection permitted minimal induction of IFN-beta. It is notable that high doses of the HCV JFH1 core protein was needed to inhibit the IPS-1-mediated IFN-beta-induction signal (Fig. 4C, left panel). Since the imaging profile of DDX3 is not always monotonous in human cells, its distribution may be biased in the cytoplasm, which may reason that only a high dose of HCV core involves preoccupied DDX3 protein to inhibit the IPS-1 pathway. This is consistent with earlier reports on an NS3-independent mechanism to block IFN induction using HCV-infected Huh 7 cells [28].

IPS-1 transduces a RNA replication signal to result in IFN-beta output using downstream proteins, such as NAP1 and IKKeysilon. If the HCV core protein interferes with IPS-1 function through DDX3, the core should not inhibit over-expressed downstream molecules. As predicted, HCV core protein did not suppress the IKKepsilon- or NAP1-mediated IFN-beta-inducing signal (Fig. 4C, center and right panels). Hence, the core protein blocks the action of endogenous DDX3 and overexpressed IPS-1 to facilitate minimal IFN-beta promoter activation, and this IFN-beta blocking function of core does not target IKKepsilon or NAP1 (Fig. 4C). An upstream molecule of IKKepsilon and NAP1 is predicted to be the target of the HCV core protein, which is in line with the fact that the HCV core protein interacts with DDX3 [14], [16].

To further confirm this model, we examined whether the HCV core protein inhibits the physical interaction between IPS-1 and DDX3. Full length IPS-1 and the C-terminal fragment of DDX3, which binds to the IPS-1 CARD-like region, were transfected into HEK293 cells, with or without the HCV core protein, and the DDX3 fragment was immunoprecipitated. Expression of HCV core proteins strongly inhibited interaction between the DDX3 C-terminal fragment and IPS-1 (Fig. 4D). JFH1 core appeared to show greater inhibition to DDX3-IPS-1 interaction than HCVO. We then examined this IFN-beta blocking function of JFH1 core in a similar cell condition plus polyU/UC. DDX3/IPS-1-enhanced p125luc reporter activity in cells stimulated with polyU/UC (Fig. 4E) was decreased in cells expressing HCV core. The results suggest that the role of the core in HCV-infected cells is to remove DDX3 from IPS-1, and facilitate its interaction with HCV replication complex (Fig. S1).

**Figure 5:**
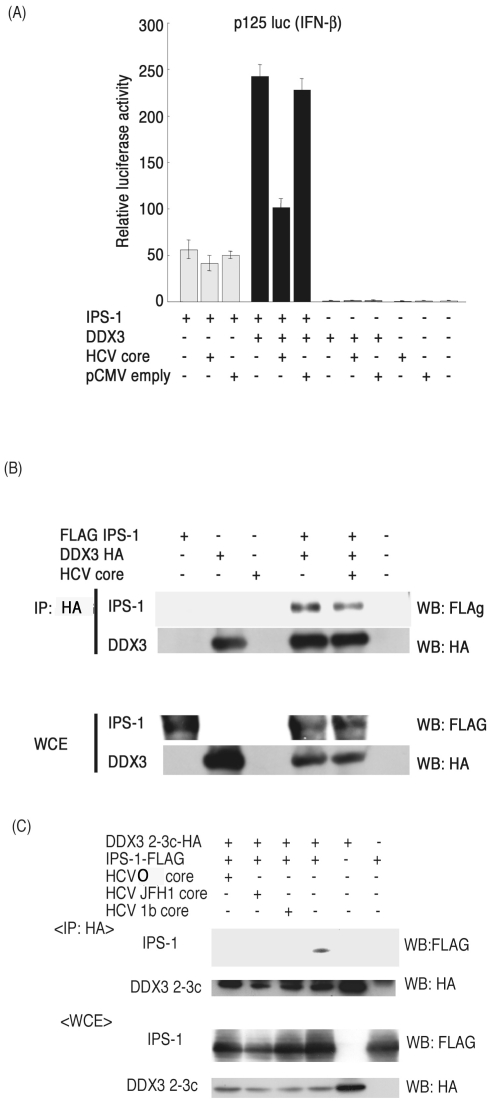
Properties of a 1b-type core protein in the IPS-1 pathway. (A) A core protein derived from an HCV patient suppressed DDX3-mediated activation of IPS-1 signaling. The 1b-type core protein was cloned into the pCMV vector from a patient with hepatitis C. IPS-1 (100 ng), DDX3 (100 ng) and HCV core (100 ng) expression vectors were transfected into HEK293 cells with a reporter plasmid (p125luc), for analysis as in Figure 4. (B) The core protein reduced interaction between full-length DDX3 and IPS-1. The plasmids encoding core protein (400 ng), DDX3-HA (400 ng) and FLAG-IPS-1 (400 ng) were transfected into HEK293FT cells. After 24 hrs, cell lysates were prepared and immunoprecipitation was carried out using anti-HA (DDX3-HA). (C) The core protein blocked interaction between the C-terminal fragment of DDX3 and IPS-1. The C-terminal region of DDX3 (199–662 aa) called DDX3 2-3c, IPS-1, HCV (O) and JFH1 or 1b core expression plasmids were transfected into HEK293FT cells. After 24 hrs, cell lysates were prepared and immunoprecipitation was carried out with anti-HA (DDX3 2–3c). Immunoprecipitates were analyzed by SDS-PAGE and Western blotting with anti-HA or FLAG antibodies. The results are representative of two independent experiments.

PolyU/UC HCV RNA activates the IFN-beta promoter (Fig. 2A), and this activity was inhibited by expression of the HCV core protein (Fig. 4E). PolyI:C/RIG-I-mediated IFN-β promoter activation was similarly suppressed by the core protein (Fig. S2A). MDA5-dependent IFN-beta promoter activation was also suppressed by the core expression (Fig. S2B). The inhibitory effect of the core protein on DDX3-IPS-1 interaction was further confirmed using an 1b core isoform isolated from a patient. This HCV core protein also reduced interaction as well as IPS-1-mediated IFN-beta promoter activation (Fig. 5A). The blocking effect was relatively weak in cells expressing IPS-1 and full-length DDX3 (Fig. 5B). We presume that this is because there are multiple binding sites for IPS-1 in the DDX3 whole molecule [11]. For binding assay, we used DDX3 2-3c (across a.a. 199∼662, longer than 224∼662) instead of the whole DDX3. In fact, DDX3(199-662)-IPS-1 interaction was blocked by the additional expression of core protein (HCVO, JFH1 or 1b core) in Fig. 5C. Ultimately, HCV core protein suppresses IPS-1 signaling by blocking the interaction between the C-terminal region of DDX3 and the CARD-like region of IPS-1, and this inhibition apparently causes the disruption of the active RIG-I/DDX3/IPS-1 complex that efficiently induces IFN-beta production signaling.

### Localization of DDX3 and HCV core protein in O cells

**Figure 6:**
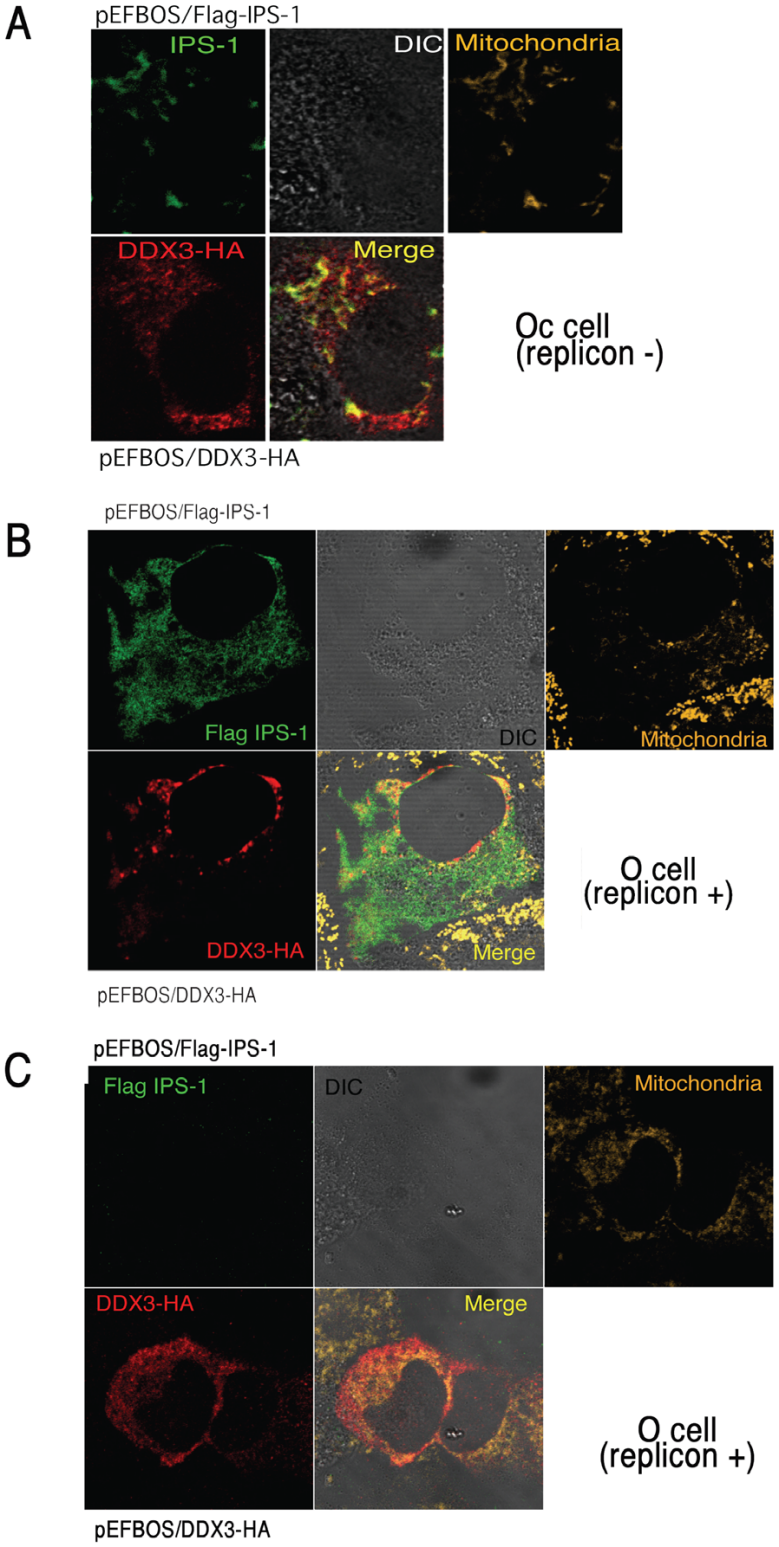
Distribution of DDX3 and IPS-1. (A) DDX3 colocalizes with IPS-1 on the mitochondria in Oc cells. HA-tagged DDX3 and FLAG-tagged IPS-1 were co-transfected into Oc cells. After 24 hrs, cells were fixed with formaldehyde and stained with anti-HA polyclonal and FLAG monoclonal Abs. Alexa488 (DDX3-HA) or Alexa633 antibody was used for second antibody. Mitochondria were stained with Mitotracker Red. Similar IPS-1-DDX3 merging profiles were observed in Huh7.5.1 cells (Fig. S3). (B,C) O cells with the HCV replicon poorly formed the DDX3-IPS-1 complex. Plasmids carrying IPS-1 (100 ng) or DDX3 (150 or 300 ng) were transfected into O (HCV replicon +) as in Oc cells (no replicon, panel A). After 24 hrs, localization of IPS-1 and DDX3 was examined by confocal microscopy. Two representatives which differ from the conventional profile (as in panel A) are shown. Similar sets of experiments were performed four times to confirm the results.

We attempted to confirm this finding by tag-expressed proteins and imaging analysis. In Huh7.5 cells IPS-1 colocalized with DDX3 around the mitochondria (Fig. S3), and so did in the hepatocyte lines Oc cells with no HCV replicon (Fig. 6A). In Oc and Huh7.5.1 cells with no HCV replicon, abnormal distribution of IPS-1 was barely observed (Fig. 6A, Fig. S3). In O cells expressing DDX3 and IPS-1, by contrast, two distinct profiles of IPS-1 were observed in addition to the Fig. 6A pattern of IPS-1: diminution or spreading of the IPS-1 protein over mitochondria (Fig. 6B,C). IPS-1 may be degraded by NS3/4A in some replicon-expressing O cells as reported previously [5], [28]. We counted number of cells having the pattern represented by Fig. 6 panel B and those similar to Fig. 6 panel C, and in most cases the latter patterns were predominant.

**Figure 7:**
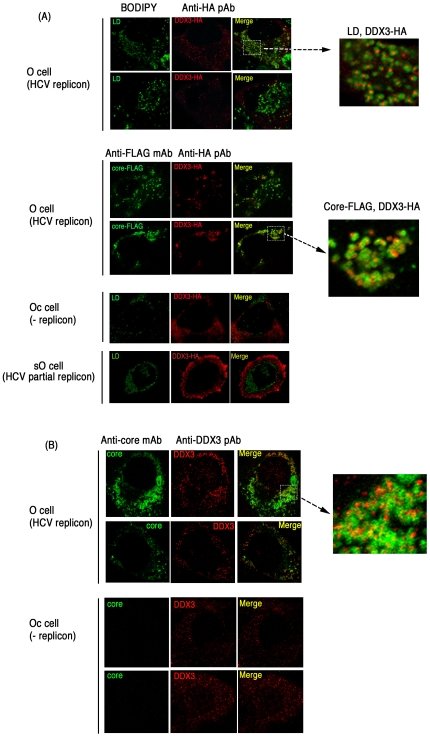
Partial association of endogenous and overexpressed DDX3 with HCV core protein in hepatocyte lines. (A) O cells with the HCV replicon form DDX3-containing speckles in the cytoplasm. O cells contain full-length HCV replicon, and Oc cells do not [16]. O cells were transfected with a plasmid expressing HA-tagged DDX3 (top panel). In other experiments, O cells were transfected with plasmids expressing HA-tagged DDX3 and FLAG-tagged HCV core protein (center panel). After 24 hrs, cells were stained with anti-HA or FLAG antibodies. Proteins were visualized with Alexa488 or 564 second antibodies and the LD was stained with BODIPY493/503. In the bottom panel, Oc cells (no replicon) and sO cells with the core-less subgenomic replicon [16] were transfected with a plasmid expressing HA-tagged DDX3. After 24 hrs, cells were stained with anti-HA antibodies. LD was stained with BODIPY493/503. (B) Endogenous DDX3-HCV core association in O cells. O or Oc cells were cultured to amplify the HCV replicon. Cells were stained with anti-core mAb and anti-DDX3pAb and secondary antibodies. Similar sets of experiments were performed three times to confirm the results.

What happens in the O cells with replicon when the core protein is expressed was next tested. Using O and Oc cells, we tested the localization of the core protein and DDX3 in comparison with IFN-inducing properties (Fig. 3). In O cells with full-length HCV replicon, DDX3 was localized proximal to the lipid droplets (LD) (Fig. 7A top panel) around which HCV particles assembled [29]. HCV core protein and DDX3 were partly colocalized in the HCV replicon-expressing cells (Fig. 7A center panel). The results were confirmed with HCV replicon-expressing O cells where endogenous core and DDX3 were stained (Fig. 7B upper panel). Partial merging between core and DDX3 was reproduced in this case, too. In contrast, sO cells, which possess a subgenomic replicon lacking the cording region of the core protein, showed no merging profile of DDX3 and LD (Fig. 7A bottom panel). Likewise, Oc cells barely formed assembly consisting of LD (where the core assembles) and overexpressed DDX3 (Fig. 7A bottom panel) or endogenous DDX3 (Fig. 7B lower panel). O cells expressing DDX3 tended to form large spots compared to Oc cells (with no replicon) and sO cells (core-less replicon) with DDX3.

Overexpressed DDX3 allowed the Oc cells to induce IPS-1-mediated IFN-beta promoter activation (Fig. 3B), while this failed to happen in O cells having HCV replicon (Fig. 3A). Ultimately, overexpressed IPS-1 did not facilitate efficient merging with DDX3 in O cells with replicon (Fig. 6B,C) compared to Oc cells or Huh7.5 cells with no replicon (Fig. 6A, Fig. S3). The results on the functional and immnoprecipitation analyses, together with the imaging profiles, infer that the IPS-1-enhancing function of DDX3 should be blocked by both NS3/4A-mediated IPS-1 degradation and the HCV core which translocates DDX3 from the IPS-1 complex to the proximity of LD in HCV replicon-expressing cells.

## Discussion

We investigated the effect of the HCV core protein on the cytosolic DDX3 that forms a complex with IPS-1 to enhance the RIG-I-mediated RNA-sensing pathway. We demonstrated that the core protein removes DDX3 from the IFN-β-inducing complex, leading to suppression of IFN-β induction. DDX3 is functionally complex, since its protective role against viruses may be modulated by the synthesis of viral proteins. DDX3 acts on multiple steps in the IFN-inducing pathway [30]. In addition, DDX3 interacts with the HCV core protein in HCV-infected cells and promotes viral replication [16]. This alternative function is accelerated by the HCV core protein, resulting in augmented HCV propagation [14], [16]. More recently, Patal et al., reported that interaction of DDX3 with core protein is not critical for the support of viral replication by DDX3, although DDX3 and core protein colocalize with lipid droplet [15]. If this is the case, what function is revealed by the interaction between DDX3 and HCV core protein remain unsettled. At least, HCV replication is not blocked by this molecular interaction [15].

It remains unclear in Fig. 4C why higher doses of JFH1 core protein are required to inhibit enhancement of IPS-1 signaling by endogenous DDX3 than by exogenously overexpressed DDX3. One possibility is that endogenous DDX3 is preoccupied in a molecular complex other than the IPS-1 pathway since DDX3 is involved in almost every step of RNA metabolism and its localization affects its functional profile [18], [30].

Together with these findings, the results presented here suggest that the HCV core inactivates IPS-1 in a mode different from NS3/4A [5], [31]. The core protein may switch DDX3 from an antiviral mode to an HCV propagation mode. The core protein localizes to the N-terminus of the HCV translation product, and is generated in infected cells before NS3/4A proteolytically liberates non-structural proteins and inactivates IPS-1. Our results on how the HCV core protein interferes with the interaction between DDX3 and IPS-1 add several possibilities to notions about the HCV function on the IFN-beta-inducing pathway [18].

DDX3 appears to be a prime target for viral manipulation, since at least three different viruses, including HCV [14], Hepatitis B virus [32], and poxviruses [8], encode proteins that interact with DDX3 and modulate its function. These viruses seem to co-opt DDX3, and also require it for replication. The viruses are all oncogenic, and may confer oncogenic properties to DDX3. DDX3 is also involved in human immunodeficiency virus RNA translocation [33]. The DDX3 gene is conserved among eukaryotes, and includes the budding yeast homolog, Ded1 [34]. The Ded1 helicase is essential for initiation of host mRNA translation, and human DDX3 complements the lethality of Ded1 null yeast [14], [35]. Another function of DDX3 is to bind viral RNA to modulate RNA replication and translocation. Constitutive expression of the HCV core or other DDX3-binding proteins may impede IFN induction and promote cell cycle progression. These reports are consistent with the implication of DDX3 in various steps of RNA metabolism in cells that contain both host and viral RNAs.

A continuing question is the physiological role of the molecular complex of DDX3 and IPS-1 during replication of HCV in hepatocytes. HCV proteins generated in host hepatocytes usually induce an HCV-permissive state in patients, for example in the IFN-inducing pathways. NS3/4A protease induces rapid degradation of IPS-1 [5], [31] and TICAM-1/TRIF [36]. NS5A interferes with the MyD88 function [37]. Viral replication ultimately blocks the STAT1-mediated IFN-amplification pathway [38]. PKR may be an additional factor by which HCV controls type I IFN production [39]. Our results add to our knowledge of the mechanism of how HCV circumvents IFN induction in host cells: HCV core protein suppresses the initial step of IFN-beta induction by interfering with DDX3-IPS-1 association. Indeed, the core protein functions as the earliest IFN suppressor, since it is generated first in HCV-infected cells, and rapidly couples with DDX3 to retract it from the IPS-1 complex, resulting in localization of DDX3 near the LD (Fig. 7). It is HCV that hijacks this protein for establishing infection. Although gene disruption of DDX3 makes mice lethal, this issue will be further tested using IPS-1 −/− hepatocytes expressing human CD81 and occludin [40], in which HCV replication would proceed.

DDX3 primarily is an accelerating factor for antiviral response through IPS-1-binding. Many host proteins other than DDX3 may positively regulate HCV replication in hepatocytes in association with the IPS-1 pathway. In this context, we know LGP2 [41] and STING [42] act as positive regulators in virus infection. Peroxisomes serve as signaling platforms for recruiting IPS-1 with a different signalosome than mitochondria [43]. It appears rational that HCV harbors strategies to circumvent these positive regulators in the relevant steps of the IFN-inducing pathway.

Imaging studies suggest that the complex of IPS-1 involving the membrane of mitochondrial/peroxisomes differ from that free from the membrane. Although IPS-1 is liberated from the membrane by NS3/4A having largely intact cytosolic domain, it loses the IFN-inducing function [5], [31]. Our results could offer the possibility that the clipped-out form of IPS-1 immediately fails to form the conventional complex for IRF-3 activation any more [44] or is easily degraded further to be inactive (Fig. 6C). Indeed, there are a number of mitochondria-specific molecules which assemble with IPS-1 [45]. Formation of the molecular complex on the mitochondria rather than simple association between IPS-1 and DDX3 may be critical for the DDX3 function.

Evidence is accumulating that HCV checks many steps in the IFN-inducing pathway throughout the early and late infection stages, and suppresses IFN production by multiple means. Disruption of IPS-1 function by both NS3/4A and core protein may be crucial in HCV-infected Huh7.5 cells, even though the cells harbor dysfunctional RIG-I [46]. Type I IFN suppresses tumors by causing expression of p53 and other tumor-suppressing agents [47]. These unique features of the HCV core protein require further confirmation, and should be minded in investigation of HCV persistency, chronic infection and progression to cirrhosis and carcinoma.

## Supporting Information

Figure S1 The IPS-1 complex. IPS-1 and HCV core bind C-terminal regions of DDX3. DDX3 captures dsRNA at the C-terminal domain. This figure is constructed from [11], [14] and [16].
(0.41 MB TIF)

Figure S2 DDX3 enhances RIG-I-mediated IFN-β promoter activation induced by polyI:C. (A) DDX3 si-1 or control siRNA was transfected into HEK293 cells with reporter plasmids and RIG-I-expression plasmid or control plasmid (100 ng). After 48 hrs, cells were stimulated with polyI:C (20 µg/ml) with dextran for 4 hrs, and activation of the reporter p125luc was measured. (B) MDA5 (25 ng), IPS-1 (100 ng), DDX3 (100 ng), JFH1 core (50 ng) and/or p125 luc reporter (100 ng) plasmids were transfected with HEK293 cells. Cell lysates were prepared after 24 hrs, and luciferase activities measured. The results are representative of two independent experiments, each performed in triplicate.
(0.17 MB TIF)

Figure S3 DDX3 colocalizes with IPS-1 on the mitochondria in Huh7.5.1 cells. HA-tagged DDX3 and FLAG-tagged IPS-1 were co-transfected into Huh7.5.1 cells. After 24 hrs, cells were fixed with formaldehyde and stained with anti-HA polyclonal and FLAG monoclonal Abs. Alexa488 (DDX3-HA) or Alexa633 antibody was used for second antibody. Mitochondria were stained with Mitotracker Red. A representative result from three independent experiments is shown.
(0.92 MB TIF)
