# Supplementary figures and images for: Hepatitis C Virus Core Protein Abrogates the DDX3 Function That Enhances IPS-1-Mediated IFN–Beta Induction
Source: PLoS One. 2010 Dec 8;5(12):e14258. doi: 10.1371/journal.pone.0014258 (PMC2999533; doi:10.1371/journal.pone.0014258)

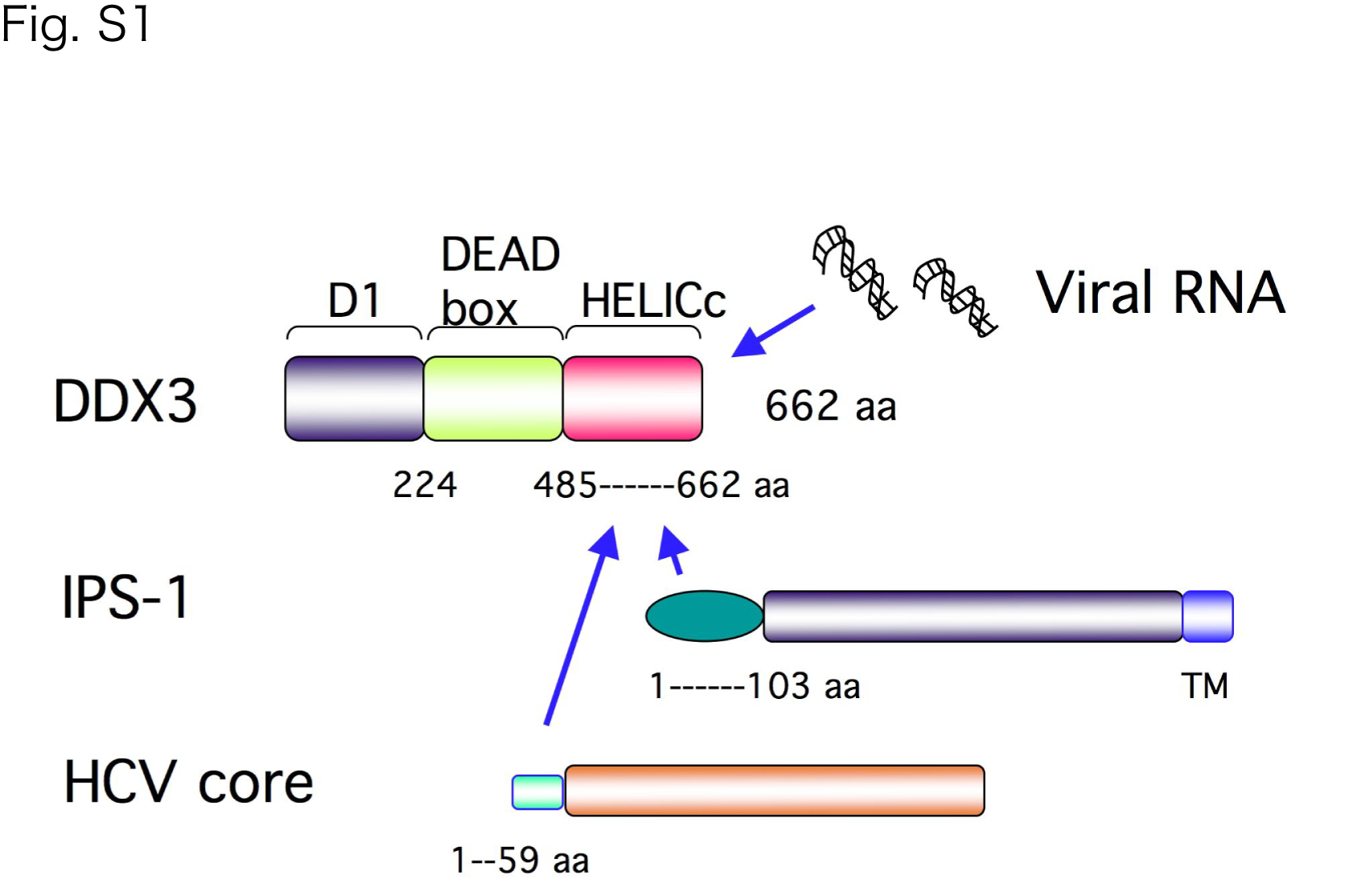

Supplement: Figure S1 — The IPS-1 complex. IPS-1 and HCV core bind C-terminal regions of DDX3. DDX3 captures dsRNA at the C-terminal domain. This figure is constructed from [11], [14] and [16]. (0.41 MB TIF) [file pone.0014258.s001.tif]

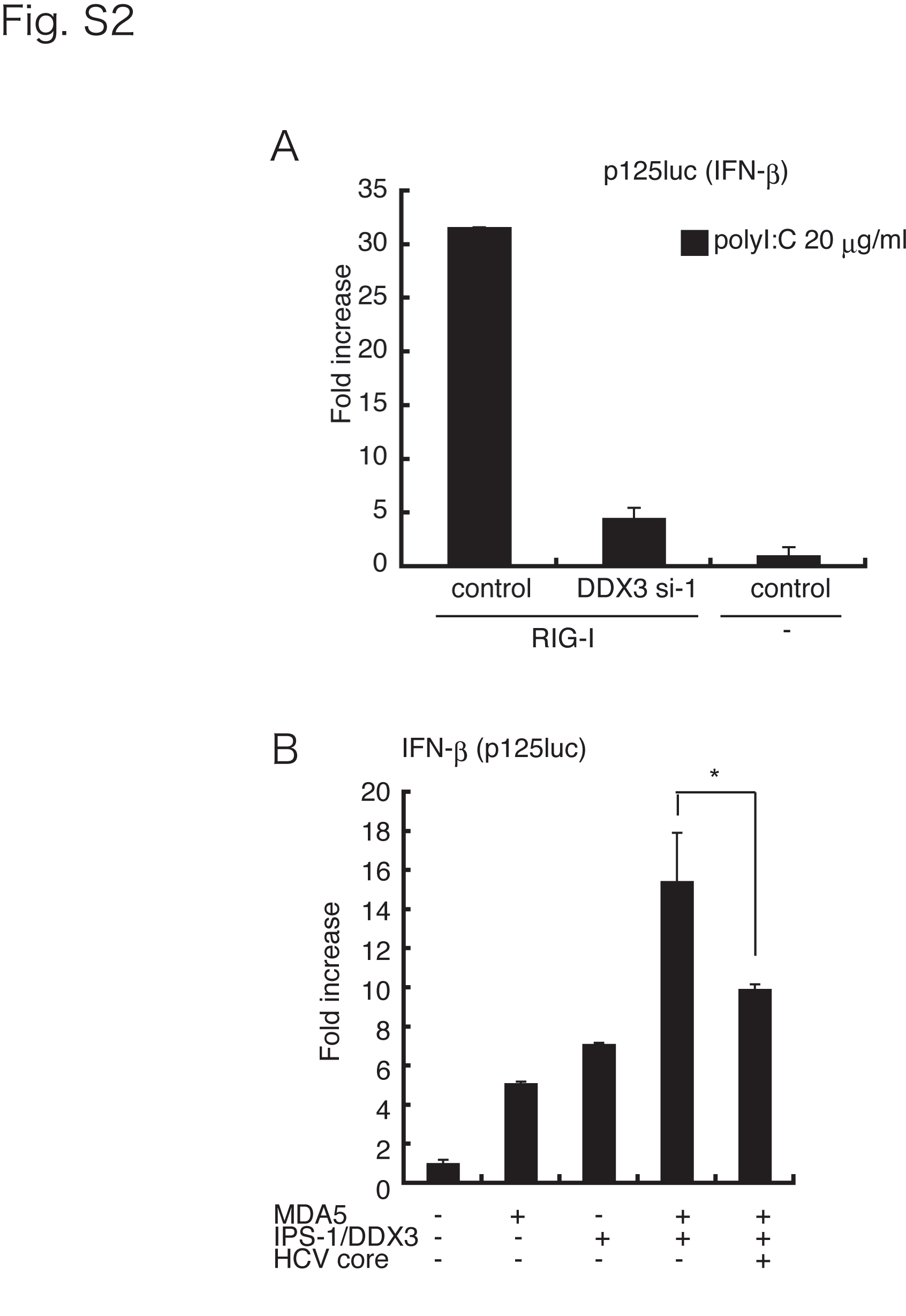

Supplement: Figure S2 — DDX3 enhances RIG-I-mediated IFN-β promoter activation induced by polyI:C. (A) DDX3 si-1 or control siRNA was transfected into HEK293 cells with reporter plasmids and RIG-I-expression plasmid or control plasmid (100 ng). After 48 hrs, cells were stimulated with polyI:C (20 µg/ml) with dextran for 4 hrs, and activation of the reporter p125luc was measured. (B) MDA5 (25 ng), IPS-1 (100 ng), DDX3 (100 ng), JFH1 core (50 ng) and/or p125 luc reporter (100 ng) plasmids were transfected with HEK293 cells. Cell lysates were prepared after 24 hrs, and luciferase activities measured. The results are representative of two independent experiments, each performed in triplicate. (0.17 MB TIF) [file pone.0014258.s002.tif]

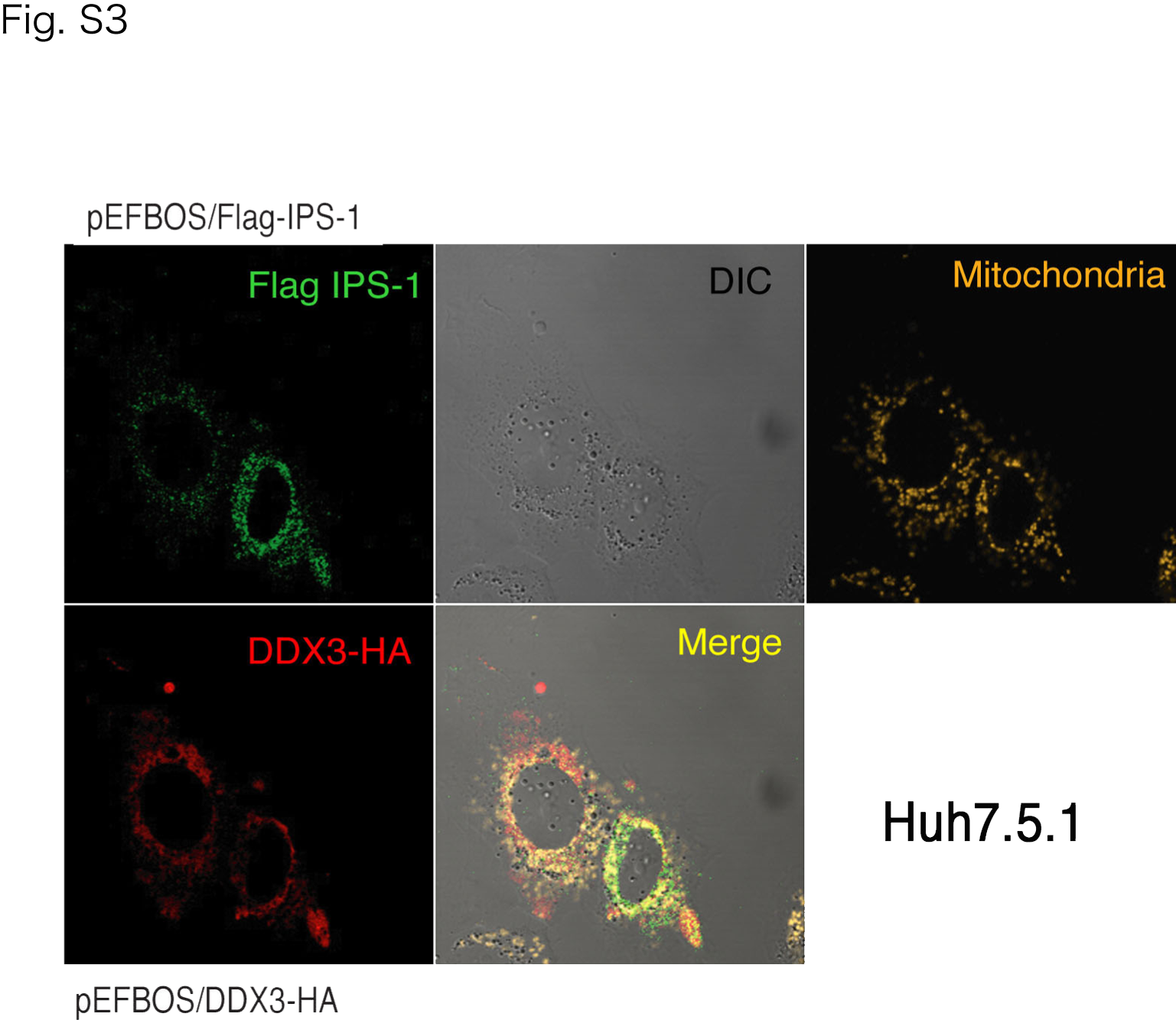

Supplement: Figure S3 — DDX3 colocalizes with IPS-1 on the mitochondria in Huh7.5.1 cells. HA-tagged DDX3 and FLAG-tagged IPS-1 were co-transfected into Huh7.5.1 cells. After 24 hrs, cells were fixed with formaldehyde and stained with anti-HA polyclonal and FLAG monoclonal Abs. Alexa488 (DDX3-HA) or Alexa633 antibody was used for second antibody. Mitochondria were stained with Mitotracker Red. A representative result from three independent experiments is shown. (0.92 MB TIF) [file pone.0014258.s003.tif]
